# A Spatial Omnibus Test (SPOT) for Spatial Proteomic Data

**DOI:** 10.1093/bioinformatics/btae425

**Published:** 2024-07-01

**Authors:** Sarah Samorodnitsky, Katie Campbell, Antoni Ribas, Michael C Wu

**Affiliations:** Public Health Sciences Division, Fred Hutchinson Cancer Center, Seattle 98109, USA; SWOG Statistics and Data Management Center, Fred Hutchinson Cancer Center, Seattle 98109, USA; Department of Medicine, Division of Hematology/Oncology, University of California Los Angeles, Los Angeles 90095, USA; Department of Medicine, Division of Hematology/Oncology, University of California Los Angeles, Los Angeles 90095, USA; Public Health Sciences Division, Fred Hutchinson Cancer Center, Seattle 98109, USA; SWOG Statistics and Data Management Center, Fred Hutchinson Cancer Center, Seattle 98109, USA

## Abstract

**Motivation:**

Spatial proteomics can reveal the spatial organization of immune cells in the tumor immune microenvironment. Relating measures of spatial clustering, such as Ripley’s *K* or Besag’s *L*, to patient outcomes may offer important clinical insights. However, these measures require pre-specifying a radius in which to quantify clustering, yet no consensus exists on the optimal radius which may be context-specific.

**Results:**

We propose a SPatial Omnibus Test (SPOT) which conducts this analysis across a range of candidate radii. At each radius, SPOT evaluates the association between the spatial summary and outcome, adjusting for confounders. SPOT then aggregates results across radii using the Cauchy combination test, yielding an omnibus *P*-value characterizing the overall degree of association. Using simulations, we verify that the type I error rate is controlled and show SPOT can be more powerful than alternatives. We also apply SPOT to ovarian and lung cancer studies.

**Availability and implementation:**

An R package and tutorial are provided at https://github.com/sarahsamorodnitsky/SPOT.

## 1 Introduction

Popular proteomic imaging platforms, such as multiplexed ion beam imaging (MIBI), multiplexed immunohistochemistry (mIHC), and imaging mass cytometry (IMC), can be used to examine the spatial distribution of cells in tissue (Wrobel *et al.* 2023). These platforms record the spatial location and protein marker expression levels—used to identify types and activities—of cells within the tissue. This permits the study of the tumor immune microenvironment (TIME), the landscape of immune cells within a tumor, at the single-cell and spatial level (Elhanani *et al.* 2023), which has been shown to be associated with clinical outcomes, such as survival (Bindea *et al.* 2013, Ali *et al.* 2016, Keren *et al.* 2018), response to treatment (Ali *et al.* 2016, Spranger 2016), and disease recurrence (Bindea *et al.* 2013).

A common analytical approach to studying the spatial distribution of cells leverages the homogeneous point process model which characterizes whether cells are randomly distributed around the image (i.e. exhibiting *complete spatial randomness* or CSR), clustered, or dispersed/repulsed (Wrobel *et al.* 2023). This model allows us to test the assumption that cells (which may be labeled by their cell type, e.g. CD8 T cell) are distributed under CSR and characterize the spatial organization of cells, offering potentially useful insights. To do so, we can summarize the spatial organization of cells within a radius *t* using spatial summary statistics like Ripley’s *K* (Ripley 1976), Besag’s *L* (Besag and Diggle 1977), and Marcon’s *M* (Marcon *et al.* 2012). Bivariate generalizations, such as bivariate Ripley’s *K*, Besag’s *L*, and Marcon’s *M*, can be used to quantify the degree of co-occurrence between two cell types and test whether two cell types are clustered together, dispersed, or randomly distributed under CSR (Wrobel *et al.* 2023).

A challenge with these spatial measures is the choice of radius, *t*, for characterizing proximal relationships. This could be guided by clinical knowledge (Barua *et al.* 2018, Masotti *et al.* 2023), but there is no consensus or guideline across applications and hypotheses. Fixing the radius at one value may neglect clinically relevant spatial patterns observed at smaller or larger values of *t*. Prior work has considered a functional analytic approach (Vu *et al.* 2022, 2023) in which spatial summary measures evaluated at multiple radii are treated as functional covariates in an outcome model. However, this requires several tuning parameters, such as the number of knots and choice of spline functions, may be computationally intensive, and may make interpretations difficult for clinicians. Alternatively, spicyR (Canete *et al.* 2022) considers a range of radii and produces an overall summary measure, termed a colocalization score, by calculating the area between the estimated spatial statistic and the theoretical value under CSR across radii, which is treated as the outcome or response variable. While this accommodates multiple radii, it does not easily accommodate censored clinical outcomes, like overall survival. Dayao *et al.* (2023) treated *t* as a tuning parameter and selected several values to be used based on the concordance index. Wilson *et al.* (2021) (as implemented in the SpatialTIME R package (Creed *et al.* 2021)) proposed a permutation-based statistic to characterize the degree of adherence or deviation from CSR. These approaches still require choosing a radius at which to interpret the results and may be challenging to synthesize an interpretation across multiple radii. This afflicts both univariate and bivariate colocalization analyses.

To address the challenge in selecting a radius, we propose an alternative approach, the SPatial Omnibus Test (SPOT). SPOT involves three steps: first, the user provides a series of radii at which to calculate a spatial summary statistic (e.g. Ripley’s *K*, Besag’s *L*); then, the association between the spatial summary and a clinical phenotype, like survival or treatment response, is tested at each radius using an appropriate model (e.g. Cox proportional hazards for a survival outcome with the spatial summary as a covariate) which results in a *P*-value describing association for each radius; finally, the *P*-values across radii are combined using the Cauchy combination test (Liu and Xie 2020). This yields a single “omnibus” *P*-value characterizing the overall strength of association between spatial organization of cells and patient outcomes. The power for detecting this association depends heavily on the user’s choice of radius, such that choosing a poor radius results in low power. On the other hand, considering multiple radii and choosing the radius resulting in greatest statistical significance leads to severe false positives. Thus, the advantages of SPOT are that, as an omnibus test, it precludes need to choose a radius *a priori*, protects the false positive rate, and maintains high power.

## 2 Materials and methods

Suppose we have *M* tumor samples, which we index by m=1,…,M. For each sample, we may have $R_{m}\geq 1$ regions-of-interest (ROIs) or images which show the spatial location of the detected cells and their phenotypes within a specific sub-region of the tumor sample. We index ROIs within a tumor sample using $r=1,\ldots ,R_{m}$. For sample *m* and ROI *r*, we assume there are *n_mr_* detected cells, irrespective of cell-type label. We index these cells using *i* and *j*, i.e. $i,j=1,\ldots ,n_{mr}$. For cell types *a* and *b*, we assume there are $n_{mr}^{a}$ and $n_{mr}^{b}$ of each in ROI *r* in sample *m*, respectively. Let $t=(t_{1},\ldots ,t_{P})$ define a vector of radii where $t_{1}=0$.

Our goal is to quantify the strength of association between the spatial organization of cells in tumors with a clinical phenotype. To do so, we follow a three-step procedure:

1. Select a series of radii, **t**, and a spatial summary measure to characterize the spatial distribution of cells. Evaluate the spatial summary measure at each radius *t_p_* for each sample *m* and ROI *r*.
2. Test the association between the spatial summary at radius *t_p_* on the clinical outcome after adjustment for clinical covariates, like age or sex, using the appropriate outcome model (e.g. Cox proportional hazards model or logistic regression). Repeat this process for $t_{1},\ldots ,t_{P}$.
3. Combine the resulting *P P*-values using the Cauchy combination test. This provides an omnibus *P*-value describing the association between the spatial summary across radii and the clinical outcome.

Next, we will describe each step to our approach in more detail. We frame our description of the SPOT method using Besag’s *L* as a spatial summary measure and survival as the clinical outcome. However, this framework is general and can accommodate many spatial summary statistics and patient outcomes best suited to the scientific question at hand.

### 2.1 Spatial summary measures

We treat the cell locations in each ROI as a spatial point pattern, which is a realization of a point process (Illian *et al.* 2008). This point process may be unmarked, meaning we disregard or do not possess additional cell-level information. This information could include functional or phenotypic marker expression, e.g. the expression of cytokeratin (CK), or categorical cell-type labels, e.g. tumor cell. We focus our discussion on categorical marks, but this framework accommodates continuous marks, as well. Note that for our purposes each point in a point pattern is a cell so we will use “point” and “cell” interchangeably.

We can describe the spatial organization of the point pattern within an ROI using second-order spatial summary statistics, which characterize the expected number of points within a radius *t*. Ripley’s *K* and Besag’s *L* are two related examples of second-order spatial summaries, which we will leverage to characterize the spatial organization of cells across tumor samples. These summary statistics are functions which take in the *n*-dimensional cell locations and output a scalar value. This value indicates how closely the point pattern adheres to the assumption of CSR, when the point (cell) locations are independent of each other. If the point pattern deviates from CSR, it may exhibit clustering or dispersion. In this case, Ripley’s *K* and Besag’s *L* quantify the degree of clustering or dispersion within the pattern.

We first define Ripley’s *K*, denoted by *K*(*t*). *K*(*t*) for a point pattern, *X*, is given by:

(1) $$\begin{array}{cc} K(t)=\frac{1}{\lambda }E(\# & \text{neighbors of}\;u\;\text{within distance}\;t\\ & \left|X\;\text{has}\;a\;\text{point at}\;u\right) \end{array}$$

where *λ* is the intensity (average number of points per unit area) of *X* assuming *X* is stationary (the properties of *X* are unchanged by a translation) and *u* is any point in the point pattern (Baddeley *et al.* 2015). Under CSR and for a given radius *t*, $K(t)=\pi t^{2}$. For a homogeneous point process, in which points or cells are equally likely to arise anywhere within the ROI, we can estimate *K*(*t*) for ROI *r* and sample *m* by:

(2) $${\hat{K}}_{mr}(t)={\hat{\lambda }}^{-1}\sum_{i=1}^{n_{mr}}\sum_{j\neq i}w_{ij}^{-1}\frac{I(d_{ij}<t)}{n_{mr}}$$

where $\hat{\lambda }=n_{mr}/A$ is an estimate of intensity, *A* is the area of the study region, *w_ij_* is an edge correction in cases when the circle of radius *t* crosses the edge of the study region, *d_ij_* is the distance between points *i* and *j*, and $I(d_{ij}<t)=1$ if $d_{ij}<t$ and 0 otherwise (Dixon 2002). In our data applications (Section 3.1 and Section 1 of the Supplementary Material), we use Ripley’s isotropic edge correction (Baddeley *et al.* 2015) to correct for edge effects. The estimate given in Equation (2) treats the cells as unlabeled. For a labeled subset of cells of type *a* (e.g. CD8 T cells only), we can adjust $\hat{K}(t)$ accordingly:

(3) $${\hat{K}}_{mr}^{a}(t)={\left({\hat{\lambda }}^{a}\right)}^{-1}\sum_{i=1}^{n_{mr}^{a}}\sum_{j\neq i}w_{ij}^{-1}\frac{I(d_{ij}<t)}{n_{mr}^{a}}$$

where ${\hat{\lambda }}^{a}=n_{mr}^{a}/A$.

Besag’s *L*, denoted by *L*(*t*), is closely related to Ripley’s *K*. Under CSR, $L(t)=\sqrt{K(t)/\pi }=\sqrt{\pi t^{2}/\pi }=t$. Our empirical estimate of *L*(*t*) for sample *m* and ROI *r* is ${\hat{L}}_{mr}(t)=\sqrt{{\hat{K}}_{mr}(t)/\pi }$. For cell type *a*, our estimate would be ${\hat{L}}_{mr}^{a}=\sqrt{{\hat{K}}_{mr}^{a}/\pi }$.

Ripley’s *K* and Besag’s *L* have bivariate generalizations to characterize the expected number of colocations between two point (cell) types. The bivariate generalization of Ripley’s *K* is:

(4) $${\hat{K}}_{mr}^{ab}(t)={\left({\hat{\lambda }}^{a}{\hat{\lambda }}^{b}A\right)}^{-1}\sum_{i}^{n_{mr}^{a}}\sum_{j}^{n_{mr}^{b}}w_{ij}^{-1}I(d_{a_{i},b_{j}}<t)$$

where $d_{a_{i},b_{j}}$ is the distance between the *i*th point of type *a* and the *j*th point of type *b*. The bivariate generalization of Besag’s *L* would then be ${\hat{L}}_{mr}^{ab}(t)=\sqrt{{\hat{K}}_{mr}^{ab}(t)/\pi }$. For the remainder of this article, we focus on Besag’s *L* as a measure of the spatial distribution of cells.

To select **t**, we use the recommended default given in the spatstatR package referred to as “Ripley’s rule-of-thumb.” This suggests using a range of radii between 0 and 0.25 times the shortest side of the image (Baddeley *et al.* 2005). In practice, some spatial summaries, particularly for small values of *t_p_*, will be 0. We suggest only including radii for which at least 20% of images have a non-zero spatial summary value. We followed this reasoning in our data applications in Section 3.1 and in Supplementary Material Section 1.

### 2.2 Outcome model

In our discussion, we focus on a survival outcome but this framework accommodates other outcome models, as well. We use a Cox proportional hazards model to examine the effect of the spatial distribution of cells or the colocalization of cell types as measured by Besag’s *L* on the log-hazard of an event. We adjust for additional clinical covariates that may confound this relationship. For a given radius, *t_p_*, define $L(t_{p})=(L_{11}(t_{p}),\ldots ,L_{1R_{1}}(t_{p}),\ldots ,L_{M1}(t_{p}),\ldots ,L_{MR_{M}}(t_{p}))$ as a vector of Besag’s *L* evaluated at radius *t_p_* across samples and ROIs. If *R_m_* = 1 for all $m=1,\ldots ,M,\;L(t_{p})=(L_{1}(t_{p}),\ldots ,L_{M}(t_{p}))$. If $R_{m}>1$ for some *m*, we average $L_{mr}(t_{p})$ within each sample *m*, i.e. $\bar{L}(t_{p})=({\bar{L}}_{1}(t_{p}),\ldots ,{\bar{L}}_{M}(t_{p}))$, where ${\bar{L}}_{m}(t_{p})=\frac{1}{R_{m}}\sum_{r=1}^{R_{m}}L_{mr}(t_{p})$. In addition to $L(t_{p})$, we may also have *B* clinical covariates contained in a design matrix X:M×B. Our Cox proportional hazards model can be specified as:

(5) $$h(s)=h_{0}(s)\;\text{exp}\;\left[L(t_{p})\beta_{t_{p}}+X\beta \right]$$

$\beta_{t_{p}}$ reflects the effect of the spatial configuration of cells or cell types within the ROIs on the log-hazard for the event-of-interest. We store the *P*-value derived from a standard Wald test of $H_{0}:\beta_{t_{p}}=0$. For each radius, $t_{1},\ldots ,t_{p}$, we obtain *P P*-values, $p_{1},\ldots ,p_{P}$.

### 2.3 Omnibus test

We have a series of *P P*-values, $p_{1},\ldots ,p_{P}$, describing the significance of the effect of $L(t_{p})$ on the log-hazard for the event. Our goal is to obtain a summary *P*-value based on $p_{1},\ldots ,p_{P}$ that describes the overall significance of the effect of Besag’s *L* across radii $t_{1},\ldots ,t_{P}$ on the log-hazard.

To obtain an overall *P*-value based on $p_{1},\ldots ,p_{P}$, we use the Cauchy combination test (Liu and Xie 2020). The Cauchy combination test was developed to combine multiple, potentially dependent, *P*-values into one summary value. For *P*-values $p_{1},\ldots ,p_{P}$, the Cauchy combination test statistic is defined as:

(6) $$T=\sum_{p=1}^{P}\omega_{p}\;\text{tan}[\pi (0.5-p_{p})]$$

where *ω_p_* represents a weight, which we fix $\omega_{p}=1/P$ for all *p*. Under the null, each $\text{tan}[\pi (0.5-p_{p})]$ follows a standard Cauchy distribution.

We use the Cauchy combination test to combine *P*-values for several reasons. We expect there is a radius or a range of radii where the association between the spatial distribution of cells and clinical outcomes is strongest. These radii are unknown *a priori* so we aggregate across several candidate radii in effort to capture this informative range. The Cauchy combination test is computationally efficient for this purpose and avoids permutation which may be required by other approaches (Wu *et al.* 2013). Permutation is challenging in this context because it requires additional computing resources and because our framework incorporates adjusting for additional sample-level covariates. Finally, the Cauchy combination test can accommodate dependent *P*-values, which is crucial in our context, because the distribution of perfectly dependent or perfectly independent Cauchy-distributed random variables is approximately the same (Liu and Xie 2020).

## 3 Results

### 3.1 Ovarian cancer application

We used SPOT to analyze images from 128 patients with high-grade serous caricoma (HGSOC) (Steinhart *et al.* 2021). The dataset was generated using the Vectra-Polaris multi-spectral immunohistochemistry (IHC) platform. We retrieved the dataset from http://juliawrobel.com/MI_tutorial/MI_Data.html but this data can also be downloaded from the VectraPolarisDataR package (Wrobel and Ghosh 2022). The detected immune cell types were B cells (cells positive for the CD19 marker or CD19+), macrophages (CD68+), CD4 T cells (CD3+, CD8−), and CD8 T cells (CD3+, CD8+). Tumor cells were identified if cells were positive for the CK marker (CK+). The cell locations were categorized as being within the tumor region or the stroma region of each ROI. For our analysis, we only considered cells located within the tumor region.

We tested for associations between the spatial configuration of each immune cell type (B cells, macrophages, CD4 T cells, CD8 T cells) with overall survival, adjusting for patient age at diagnosis. We also tested for an association between spatial colocalization of each pair of immune cells with overall survival, adjusting for patient age. Within both analyses, we adjusted the *P*-values for multiple testing using an FDR adjustment (the adjustment was applied over four tests in the first analysis and six tests in the second analysis). Since the dimensions of the images varied, we choose a range of radii between 0 and 0.25 times the smallest image width across the images which was 0.25*1009.6=252.4. We considered 100 different radii between 0 and 252.4.

We found that the clustering of each immune cell type individually was not associated with overall survival. The FDRs for each cell type were *P *=* *.261 for macrophages, *P *=* *.486 for B cells, *P *=* *.477 for CD8 T cells, and *P *=* *.936 for CD4 T cells. Within a short window ($t_{p}\in (2.55,12.75)$), the association between macrophage clustering and overall survival appeared significant but dissipated as the radius increased.

**Figure 1.**
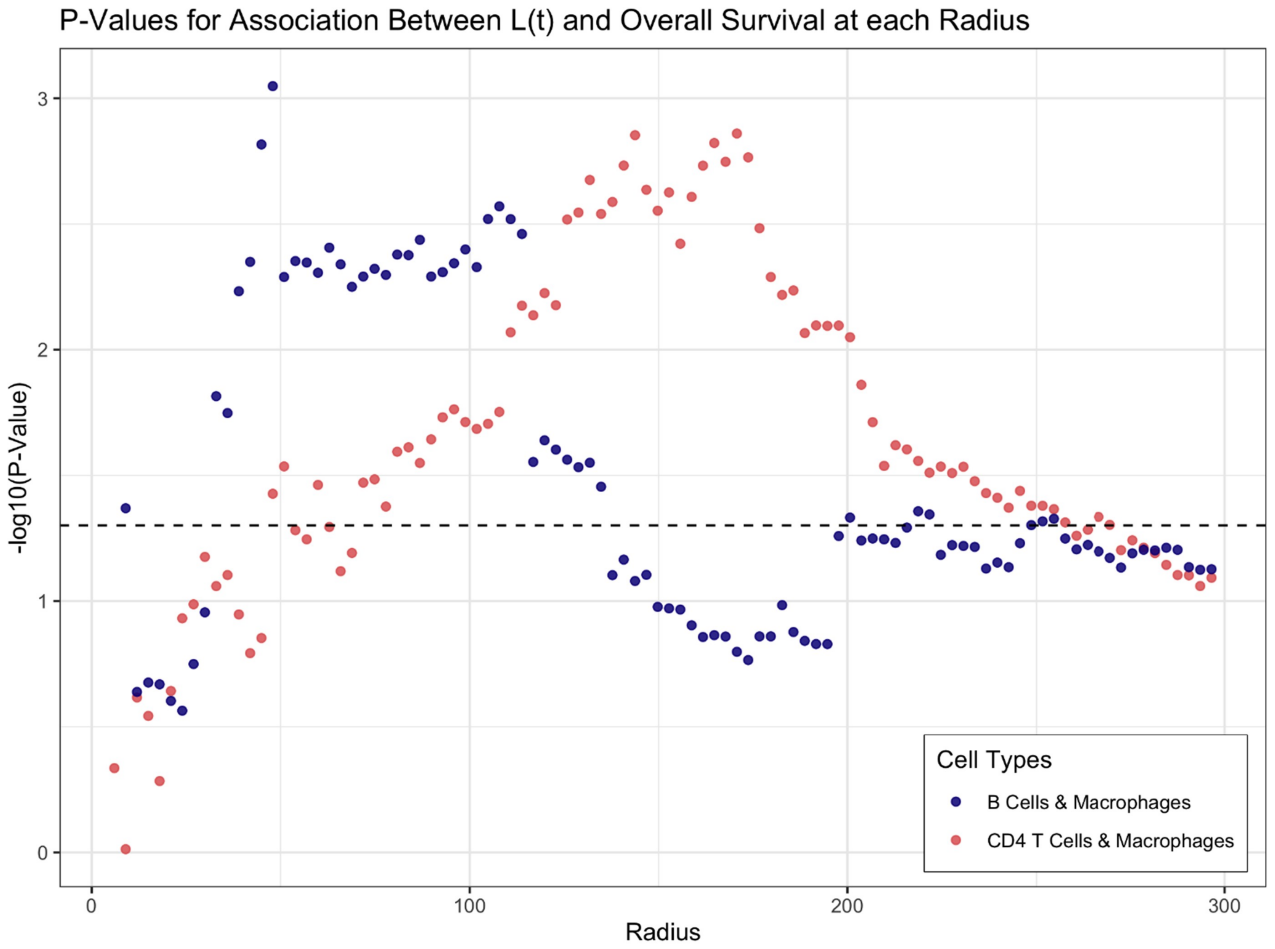
The relationship between the radius, *t*, and the *P*-value for the effect of CD4 T cell-macrophage colocalization and macrophage-B cell colocalization as measured by *L*(*t*) on overall survival. This shows a plausible range of radii where an association between CD4 T cell-macrophage colocalization and survival is observable. Note that these *P*-values are not adjusted for multiple testing and should not be interpreted in the traditional sense to avoid inflating type I error.

We then tested the association between the colocalization of pairs of immune cells and overall survival. We found that the colocalization of CD4 T cells and macrophages was significantly associated with overall survival (FDR =0.0286). We also found that the colocalization of macrophages and B cells was significantly associated with overall survival (FDR =0.0286) (Table 1, Fig. 1). These results align with Steinhart *et al.* (2021)’s analysis of this dataset, which revealed that proximity between macrophages and B cells and between macrophages and CD4 T cells was associated with overall survival.

**Table 1.** Association between the colocalization of each immune cell type pair and overall survival in ovarian cancer.

Cell type 1	Cell type 2	SPOT *P*-value	FDR
CD4 T cell	Macrophage	.0071	0.0286
Macrophage	B cell	.0095	0.0286
CD4 T cell	CD8 T cell	.1887	0.3774
Macrophage	CD8 T cell	.3959	0.5938
CD8 T cell	B cell	.9191	0.9216
CD4 T cell	B cell	.9216	0.9216

### 3.2 Simulation studies

#### 3.2.1 Power at each fixed radius

We first evaluated the power of testing the association between a spatial summary at a series of individual radii with survival against SPOT’s power. For this simulation, we used Besag’s *L* as our spatial summary measure. We generated images for *M *=* *100 samples. To generate a survival outcome, we simulated the event times for the first 50 samples from Exponential(λ=log(2)/12) (the low-survival group) and the event times for the last 50 samples from Exponential(λ=0.4 log(2)/12) (the high-survival group), where *λ* represents the rate or hazard parameter. We randomly censored 10–20% of event times in each group. Each image was generated to have dimension 1000 × 1000. We generated between 50 and 100 cells in each image. We generated the cell locations for each image under two conditions: (1) exhibiting CSR or (2) exhibiting spatial clustering. To generate an image under CSR, we simulated the (*x*, *y*) coordinates for each cell from a uniform distribution, i.e. x,y∼Uniform(0,1000). To generate a clustered image, we first simulated mean *x* and *y* locations from a uniform distribution, i.e. $\mu_{x},\mu_{y}∼\text{Uniform}(100,900)$, and then simulated the (*x*, *y*) coordinates for each cell from a multivariate-normal distribution: $\left(x,y)∼\text{Multivariate-Normal}({\left(\mu_{x},\mu_{y}\right)}^{T},\Sigma \right)$, where $\Sigma =\left(\begin{array}{cc} 100^{2} & 50*100\\ 50*100 & 100^{2} \end{array}\right)$. For one cell type, the point pattern was treated as unmarked. For two cell types, each point location was randomly assigned a cell-type label so that there were approximately equal numbers of each type.

To estimate power, we simulated images for the high-survival group to be uniform and images for the low-survival group to be clustered. We considered the power at each radius value from 0 to 250. We ran the simulation for 1000 replications and calculated power using the proportion of simulation replications the *P*-values were below a significance level of .05.

**Figure 2.**
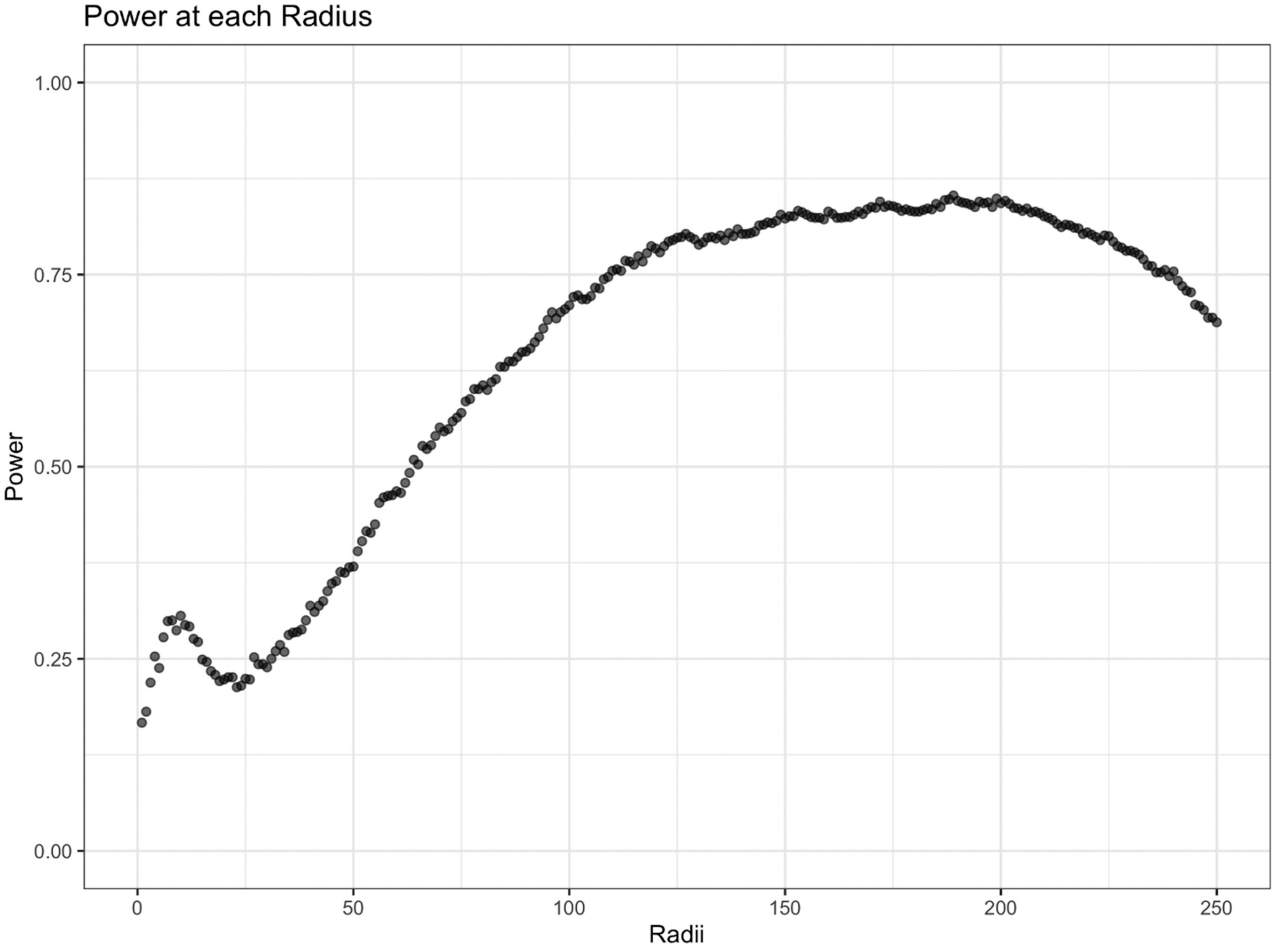
The power of testing the association between Besag’s *L* at each radius and survival.

The results are shown in Fig. 2. As the radius increases, we observe a gain in power across the radii before a slight descent. The maximum individual power was 0.853 at a radius of 189 whereas SPOT’s power was 0.867. This illustrates the variability in power observed at each radius and the challenge of choosing the “best” radius at which to relate the spatial distribution of cells with clinical outcomes.

#### 3.2.2 Methods comparison

We then compared SPOT to existing methods. We considered SPF (Vu *et al.* 2022) and FunSpace (Vu *et al.* 2023) as alternative approaches. These methods allow for a range of radii and treat the spatial summary measure evaluated at each radius as a functional covariate. We also compared these methods against a “naive” approach in which we select the “best” and “worst” radii at which to calculate a spatial summary. For SPOT, SPF, FunSpace, and the naive approach, we use Besag’s *L* as our spatial summary statistic. We compared the approaches in terms of their type I error rate and power across several conditions. Throughout the simulation, we varied the:

- Outcome type (survival or binary)
- Number of cell types (one or two)
- Number of images per sample (one or multiple)
- Clustering pattern (multivariate normal, Matérn clustering, Strauss process)
- Sample size (*M* = 100 or M=30)

Here, we focus on the results for a survival outcome, a single image per sample, *M *=* *100, and analyzing images with cell locations simulated from either a multivariate Gaussian distribution or under CSR. Additional simulation conditions and examples of the simulated images are shown in Supplementary Material Section 2. We generated images of dimension 1000 × 1000 and a survival outcome in an analogous manner as described in Section 3.1.

We fit SPF and FunSpace in the following manner. To fit SPF, we used Besag’s *L* as the spatial summary measure for both one and two cell types. When we generated multiple images per sample, we averaged the Besag’s *L* output across ROIs within a sample. We also extended SPF to allow for a binary outcome. We also fit FunSpace with the mxfda R package (Wrobel and Soupir 2024) available at https://github.com/julia-wrobel/mxfda using Besag’s *L* for one and two cell types and extended FunSpace to allow for a binary outcome.

To estimate the type I error rate, images were randomly generated to be uniform or clustered. To estimate power under conditions where each sample had only one image, we simulated images for the high-survival group to be uniform and images for the low-survival group to be clustered. To estimate power under conditions in which there were multiple images per sample, we simulated all images to be uniform for the high-survival group. For the low-survival group, we randomly chose some images to be clustered and some to be uniform. The probability of an image being generated as clustered in this group was 0.75. We calculated type I error and power using the proportion of simulation replications the *P*-values were below a significance level of .05.

We ran each simulation condition for 1000 replications. The results for one image per sample are shown in Table 2. With both one and two cell types, SPOT exhibits the lowest type I error rate (one cell type: 0.057 for SPOT vs. 0.061 for SPF and 0.124 for FunSpace; two cell types: 0.054 vs. 0.061 for SPF and 0.139 for FunSpace). At the “best” radius, the power for one cell type was 0.992 and 0.991 for two cell types, though the type I error rate was 0.456 for one cell type and 0.483 for two cell types. At the “worst” radius, the power for one cell type was 0 and 0.001 for two cell types, though the type I error rate was 0.006 for one cell type and 0.005 for two cell types. This illustrates that SPOT provides a middle-ground between choosing the “best” or ideal radius in every condition, at which the type I error rate is high, and the “worst” or most-conservative radius, at which power is very low.

**Table 2.** Type I error rates and power with a survival outcome and one image per sample.

	One cell type		Two cell types	
Model	Type I error rate	Power	Type I error rate	Power
SPOT	0.057	0.925	0.054	0.867
FunSpace	0.124	0.714	0.139	0.621
SPF	0.061	0.862	0.061	0.770
Choose “best” radius	0.456	0.992	0.483	0.991
Choose “worst” radius	0.000	0.006	0.001	0.005

## 4 Discussion

We proposed a SPOT for the association between spatial summary measures of cell organization in the TIME with clinical outcomes, like survival or treatment response. These summary measures typically require the user to select a radius at which to characterize spatial organization of cells. However, there is no rule-of-thumb or guideline for making this selection across applications. SPOT provides a straightforward framework for relating the cellular architecture (or cell type colocalization) with outcomes across multiple radii. We found *via* simulation that SPOT provides a reasonable middle-ground between choosing the “best” radius, which is difficult to know *a priori* and may lead to false positive discoveries, and the “worst” radius, which offers very little power. We also applied SPOT to an ovarian cancer dataset, which corroborated the prognostic importance of CD4 T cell and macrophage colocalization, as well as macrophage and B cell colocalization (Steinhart *et al.* 2021).

The advantage of the SPOT framework is that it is adaptable to the application and hypotheses of practitioners. For example, one could consider any measure of spatial cellular configuration, such as the mark connection function used by Vu *et al.* (2022) or estimate the spatial intensity of cells and consider the Jensen−Shannon distance between density estimates as done in Masotti *et al.* (2023). One could consider spatial summary measures that accommodate inhomogeneity and could implement any outcome model depending on the clinical outcome-of-interest. Further, SPOT could easily be parallelized across radii to improve computation speed.

We used Ripley’s rule-of-thumb for determining radii ranges. Our data analyses suggest that this range is reasonable, but one could expand beyond this. Other approaches for choosing the radii may incorporate biological knowledge, such as the size of the cell and prior knowledge about the “radii-of-influence” between cell types (Masotti *et al.* 2023). Consideration of additional radii can boost power if useful radii are included, but consideration of poor choices can lead to reduced power. Thus, if one has prior contextual knowledge, applying SPOT to a more targeted, reduced set of radii may offer improvement.

We emphasize that we focused on Besag’s *L*, but SPOT can be applied to any relevant and valid summary statistic for convenience. The framework remains the same, simply substituting the choice of metric. In principle, we could further extend SPOT to simultaneous consideration of multiple metrics, as well as radii, but this remains a potential topic for further investigation. Finally, we did not address the issue of holes or gaps in the image that may arise in tumor resections using spatial proteomics imaging platforms. The challenge of gaps in the image is that it violates the assumption of homogeneity among the points in a spatial point pattern. One approach to address this is to incorporate a simulation envelope in which the cell-type labels are permuted (Creed *et al.* 2021, Wilson *et al.* 2021, Seal *et al.* 2024). This approach could be incorporated into SPOT, though the computational cost remains high. As further approaches are developed in this area, we anticipate their potential incorporation into our proposed framework and the continued growth of our method.

## Supplementary Material

btae425_Supplementary_Data

## Data Availability

Ovarian and non-small cell lung cancer datasets are available from http://juliawrobel.com/MI_tutorial/MI_Data.html. Lung.
