# Supplementary material for: A Spatial Omnibus Test (SPOT) for Spatial Proteomic Data
Source: Bioinformatics. 2024 Jul 1;40(7):btae425. doi: 10.1093/bioinformatics/btae425 (PMC11257711; doi:10.1093/bioinformatics/btae425)
Supplement: btae425_Supplementary_Data [file btae425_supplementary_data.pdf]

Supplementary Materials: Spatial Omnibus Test (SPOT) for Spatial Proteomic Data

Sarah Samorodnitsky, Katie Campbell, Antoni Ribas, Michael C. Wu

1 Non-Small Cell Lung Cancer Application

We used SPOT to analyze multiplexed immunohistochemistry images obtained from $n = 153$ resections from non-small cell lung cancer tumors [1]. The dataset is publicly available from `http://juliawrobel.com/MI_tutorial/MI_Data.html`. Between four and six regions-of-interest (ROIs) were available per sample for a total of 761 images. There were five detected cell types in the dataset: CD14+ cells, CD19+ B cells, CD4+ T cells, CD8+ T cells, and CK+ tumor cells. All other cells types were labeled as "Other." Samples were categorized based on if the tumor cells exhibited high major histocompatibility complex II (MHCII). Samples were given this label if more than 5% of tumor cells expressed MHCII.

We were interested in relating the spatial distribution of immune cells to binary MHCII status of the tumor. We considered the spatial distributions of each individual immune cell (CD14+ cells, CD19+ B cells, CD4+ T cells, and CD8+ T cells) and the

colocalization of each immune cell type with tumor cells. To accommodate multiple ROIs, we averaged Besag's L values across ROIs within a sample. We considered a sequence of 100 radii between 0 and 125.25 following Ripley's rule-of-thumb. To address multiple testing, we controlled the Benjamini-Hochberg false discovery rate (FDR) [2].

We did not find any significant associations between the spatial distribution of individual immune cells and high MHCII status. We did, however, find significant associations between the colocalization of each immune cell type with tumor cells at the FDR 0.1 level. The FDRs for were: 0.0677 for CD4+ T cells and tumor cells, 0.0677 for CD14+ cells and tumor cells, 0.0677 for CD19+ B cells and tumor cells, and 0.0894 for CD8+ T cells and tumor cells (Figure 1). This aligns with Johnson et al.'s findings, which showed that tumor cells colocalized more with CD4+ and CD8+ T cells in MHCII-high tumors [1].

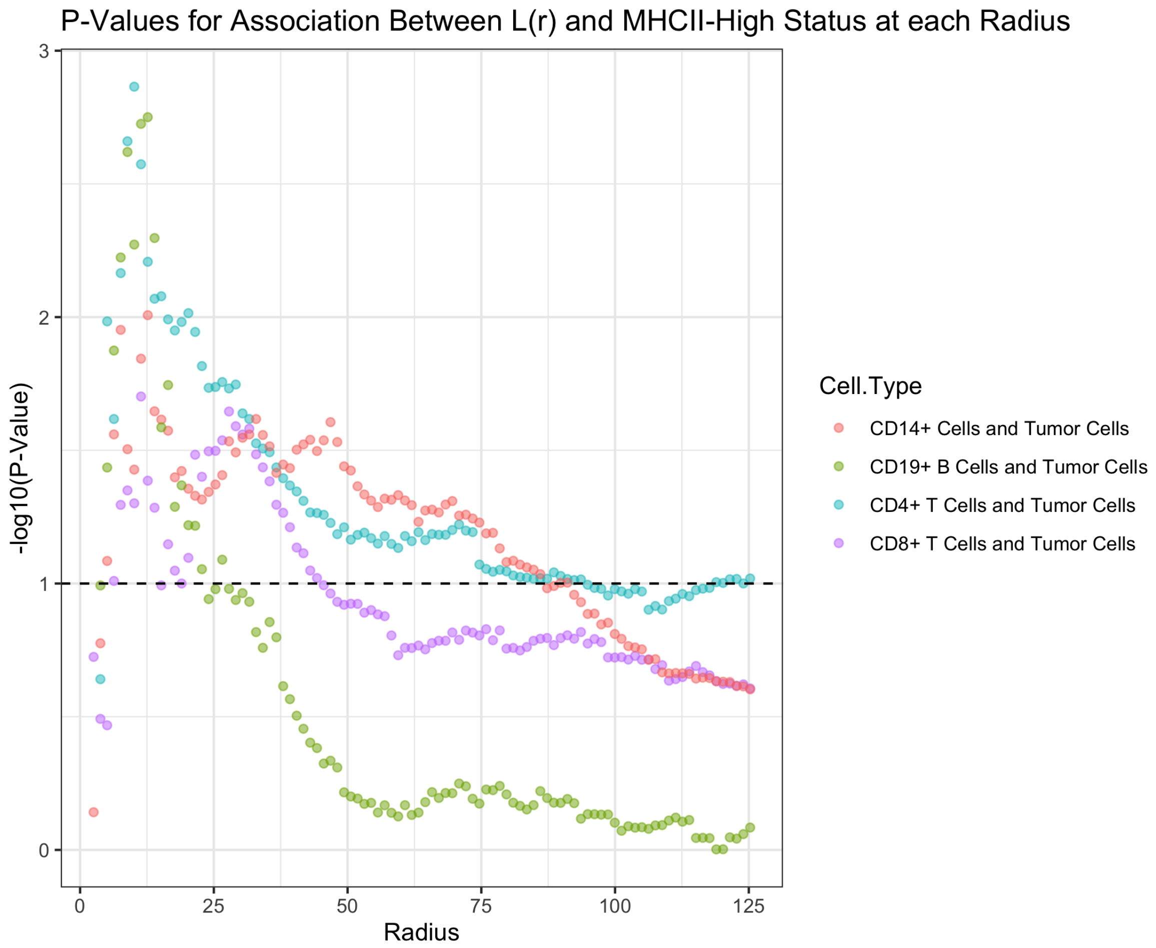

Figure 1: P-value trajectories for the association between the spatial colocalization of each immune cell type and tumor cells with MHCII-high status. Dashed line indicates a significance level of 0.1. Note that these p-values have not been adjusted for multiple comparisons and should be used only to gauge radii ranges where an association between cell type colocalization and outcome exists. The p-values should not be interpreted in the traditional sense to avoid inflating type I error.

2 Simulation Studies

2.1 Simulation Conditions

Here we provide examples of the types of images generated throughout our simulation study. All images were generated to have dimensions 1000×1000. In the simulation results described in the main manuscript, we generated cell locations either from a multivariate normal distribution or a uniform distribution. The number of cells was sampled randomly between 50 and 100. For the multivariate normal distribution, we first simulated means, μ_x and μ_y, in the x- and y-directions uniformly between 100 and 900. Cell locations were then simulated from $(x, y) \sim \text{Multivariate-Normal}((\mu_x, \mu_y)^T, \mathbf{\Sigma})$ where $\mathbf{\Sigma} = \left(\begin{smallmatrix} 100^2 & 50*100 \\ 50*100 & 100^2 \end{smallmatrix}\right)$.

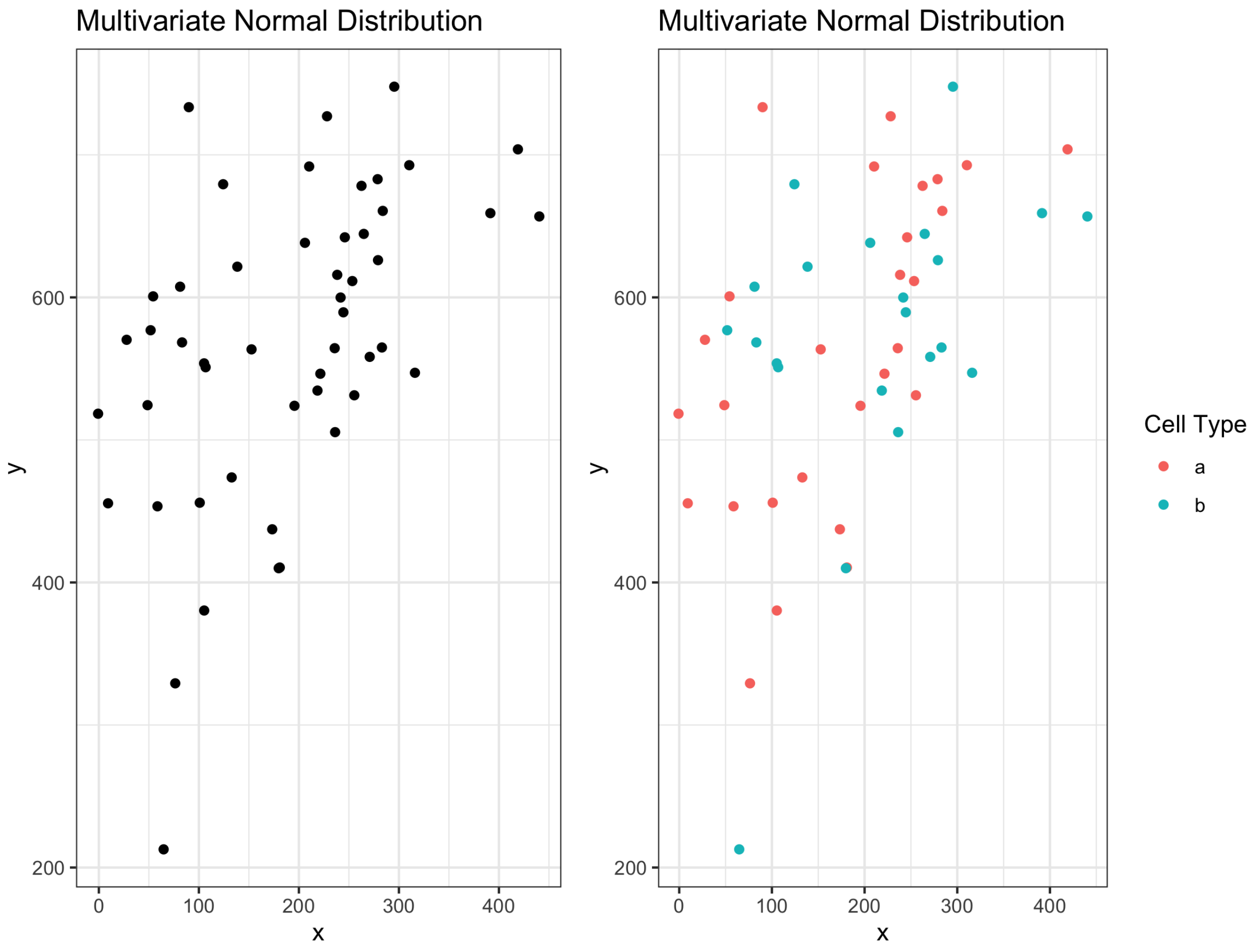

Figure 2: Example images for simulating cell locations from a multivariate normal distribution with either one cell type or two.

For the uniform distribution, we simulated the x- and y-coordinates of each cell's location from a uniform distribution between 0 and 1000 (Figure 3).

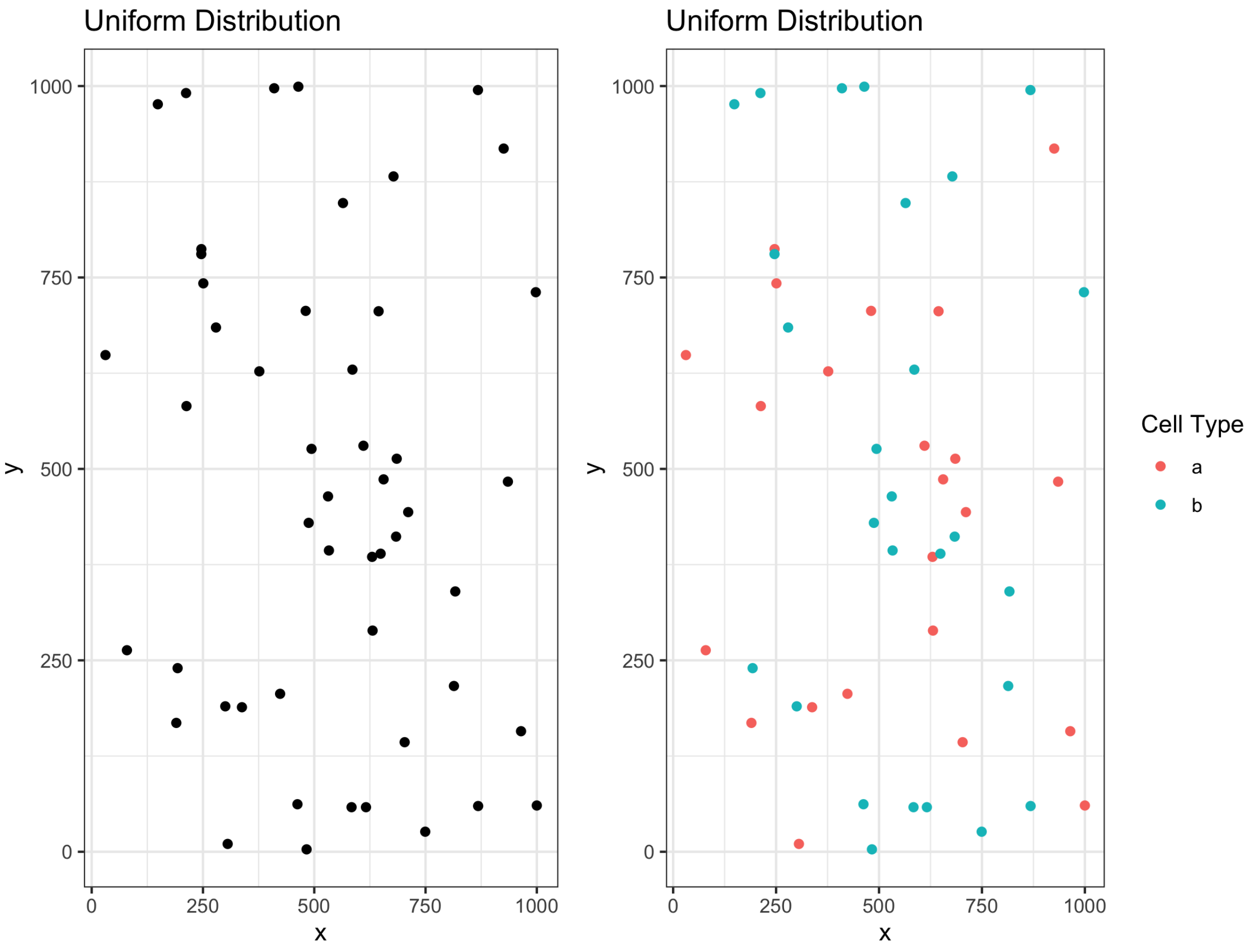

Figure 3: Example images for simulating cell locations from a uniform distribution with either one cell type or two.

We also considered simulating cell locations from a Matérn cluster process or a Strauss process [3]. In Section 2.4, we describe results under two conditions where we split the images into two groups and simulated cell locations from: (1) either a Matérn clustering process or under CSR and (2) either a Strauss process or under CSR. The number of cells was determined by the corresponding process. For the Matérn cluster process, we used an intensity of 0.00001, fixed the radius of each

cluster at 10, and the average number of cells per cluster at 15 (Figure 4).

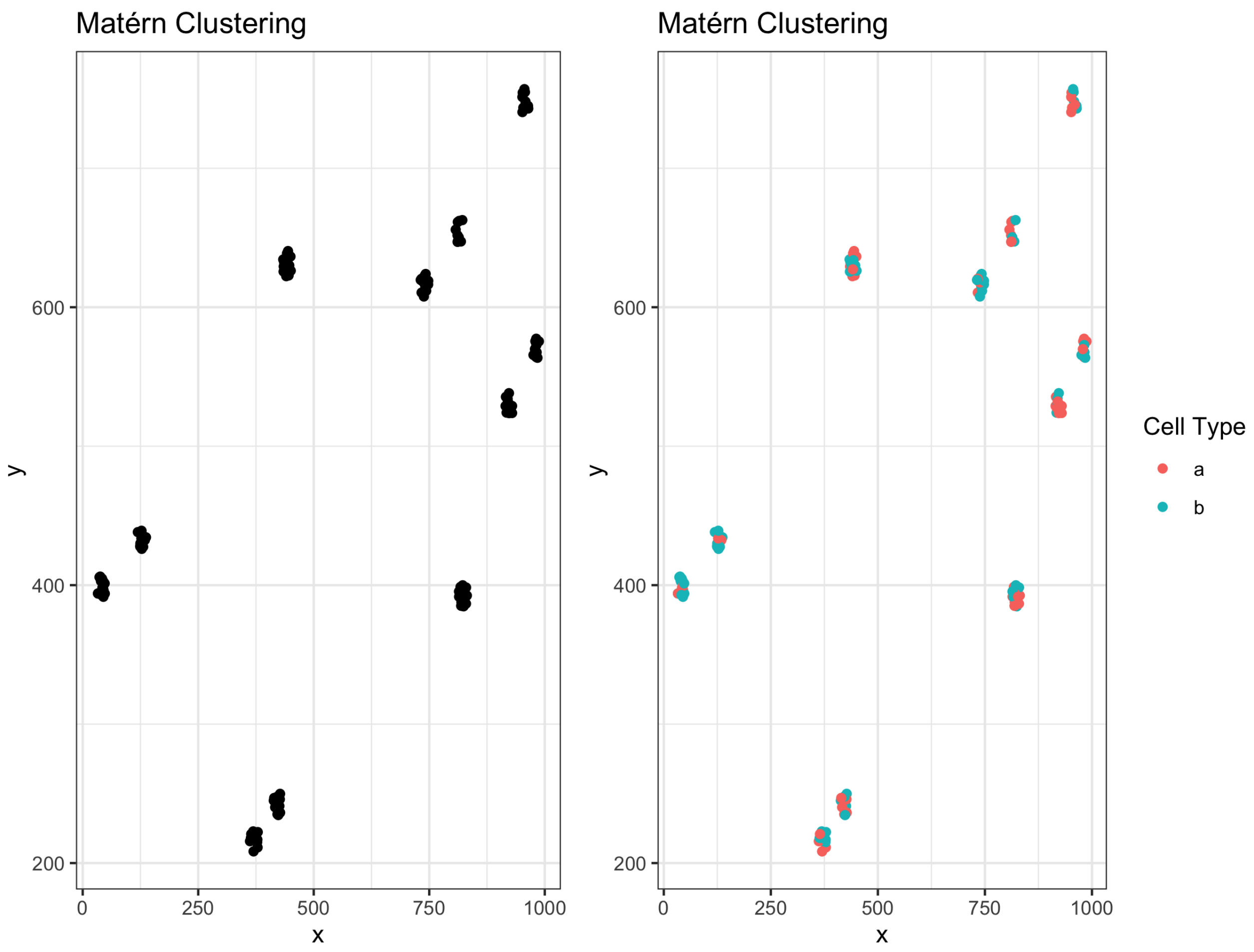

Figure 4: Example images for simulating cell locations from a Matérn cluster process with either one cell type or two.

For the Strauss process, we used an intensity of 0.0001, an interaction parameter of 0, and an interaction radius of 50. We fixed the interaction parameter at 0 to ensure points were no closer than 25 units apart, as determined by half our interaction radius, to mimic repulsion among the cell locations (Figure 5).

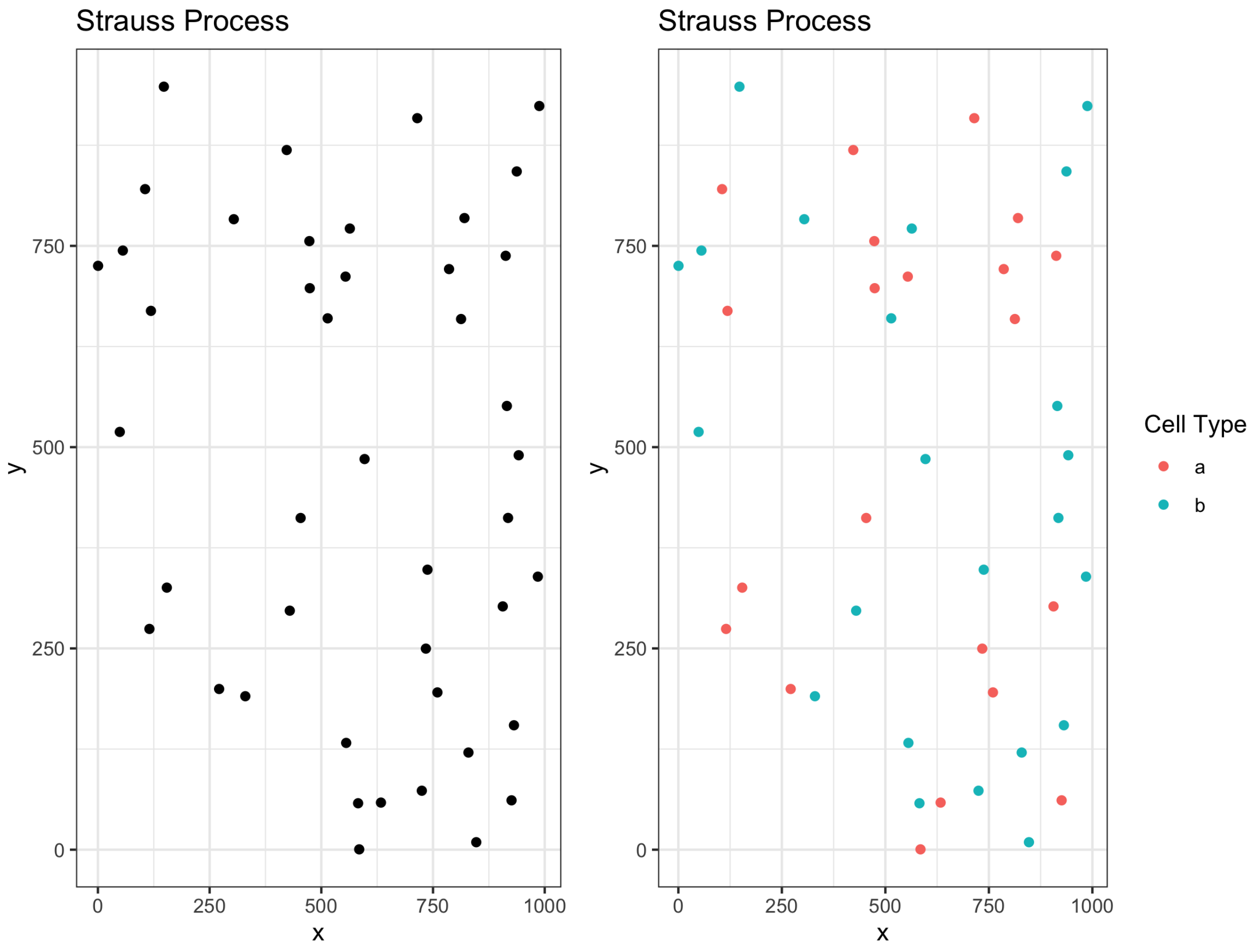

Figure 5: Example images for simulating cell locations from a Strauss process with either one cell type or two.

2.2 Binary Outcome

Here we consider the performance of SPOT against existing methods for relating spatial summary measures to a binary outcome with a single image per sample. We simulated images as described in Section 4 of the main manuscript (i.e. samples

were split into two groups and images were simulated from either a multivariate normal distribution or under CSR). We compared SPOT to SPF and FunSpace, as considered in the main manuscript, as well as SpaceANOVA [4] and spicyR [5]. SPF and FunSpace were not originally developed for a binary outcome. We extended these methods to leverage a functional logistic regression model. SpaceANOVA is another functional data analytic approach that treats a spatial summary measure (we use the g-function) evaluated across radii as a functional outcome within a functional ANOVA model. spicyR evaluates a spatial summary statistic (we use Besag's L) across a range of radii and computes the area between the empirical and theoretical spatial summary. This summary value is treated as an outcome in a linear model. We fixed the radii considered in SPF, FunSpace, SpaceANOVA, and spicyR at the same values considered for SPOT, which were from 0 to 250 by increments of 1.

The results for one image per sample are shown in Table 1. For both one and two cell types, SPOT controls type I error and offers power between 0.89 and 0.96, though SpaceANOVA provides the highest power.

	1 Cell Type		2 Cell Types	
**Model**	**Type I Error Rate**	**Power**	**Type I Error Rate**	**Power**
SPOT	0.042	0.960	0.054	0.889
FunSpace	0.112	0.619	0.148	0.536
SPF	0.034	0.822	0.042	0.653
SpaceANOVA			0.062	0.999
spicyR	0.040	0.741	0.056	0.617
Choose 'Best' Radius	0.396	0.999	0.465	0.991
Choose 'Worst' Radius	0.000	0.007	0.000	0.001

Table 1: Type I error rates and power for a binary outcome and one image per sample.

2.3 Multiple Images

We now describe our simulation results when we considered a survival and a binary outcome and multiple images per sample. Images were generated as discussed in Section 4 of the main manuscript. Briefly, to estimate power, we simulated all images to be uniform for the high-survival group. For the low-survival group we randomly chose some images to be clustered using a multivariate Gaussian distribution and some to be uniform. The probability of an image being generated as clustered in this group was 0.75. We randomly chose the number of images to simulate for each sample between one and three. For a survival outcome, we compared SPOT to SPF. For a binary outcome, we compared SPOT to SPF, SpaceANOVA, and spicyR. To

accommodate multiple images, SpaceANOVA and spicyR both incorporate a random effect. For SPF, we average the spatial summary statistic across ROIs within a sample. We were unable to accommodate FunSpace under this condition.

The results for a survival outcome shown in Table 2. With one cell type, SPOT exhibited slightly elevated type I error rates (0.065). This may be because the Cauchy combination test better controls type I error at lower significance levels. With two cell types, SPOT controlled type I error around 0.053 and exhibited much higher power than SPF (0.872 vs. 0.772). As discussed in the main manuscript, choosing the "best" radius yielded inflated type I error rates and the "worst" offered very little power. Similar to our results for a single image per sample, SPOT offers a balance between the "best" and "worst" radius in providing nominal type I error rates and adequate power.

	**1 Cell Type**		**2 Cell Types**	
**Model**	**Type I Error Rate**	**Power**	**Type I Error Rate**	**Power**
SPOT	0.065	0.921	0.053	0.872
SPF	0.055	0.857	0.046	0.772
Choose 'Best' Radius	0.471	0.991	0.492	0.985
Choose 'Worst' Radius	0.000	0.003	0.000	0.008

Table 2: Type I error rates and power with a survival outcome and multiple images per sample

The results for a binary outcome are shown in Table 3. SPOT controls type I error and provides adequate power. SpaceANOVA excels at controlling type I error

and power under this condition.

Model	1 Cell Type		2 Cell Types	
	**Type I Error Rate**	**Power**	**Type I Error Rate**	**Power**
SPOT	0.037	0.940	0.046	0.856
SPF	0.024	0.795	0.039	0.634
SpaceANOVA			0.048	1.000
spicyR	0.039	0.729	0.051	0.631
Choose 'Best' Radius	0.426	1.000	0.472	0.987
Choose 'Worst' Radius	0.000	0.004	0.000	0.003

Table 3: Type I error rates and power for a binary outcome and multiple images per sample.

2.4 Other Clustering Patterns

Here we examine SPOT's performance on two additional clustering patterns: Matérn clustering process and Strauss process. As described in Section 4 of the main manuscript, samples were split into two groups. In one group, images were simulated under CSR. In the other group, images were simulated either from a Matérn clustering process (Figure 4) or from a Strauss process (Figure 5) as described in Section 2.1. We considered $M = 100$ samples, each with a single image. Survival outcomes were simulated as described in the main manuscript. The results for Matérn clustering given in Table 4. SPOT, FunSpace, and SPF all exhibited comparable type I error

rates and power. For two cell types, SPOT and FunSpace best controlled type I error and SPF offered a slight advantage in power.

	**1 Cell Type**		**2 Cell Types**	
**Model**	**Type I Error Rate**	**Power**	**Type I Error Rate**	**Power**
SPOT	0.063	0.984	0.052	0.979
FunSpace	0.061	0.942	0.053	0.936
SPF	0.062	0.978	0.063	0.982
Choose 'Best' Radius	0.233	0.999	0.219	0.996
Choose 'Worst' Radius	0.000	0.026	0.001	0.033

Table 4: Type I error rates and power for a survival outcome with a single image per person and cell locations simulated using a Matérn cluster process.

The results for images generated using a Strauss process are shown in Table 5. In this case, SPOT provided the best type I error control and the highest power, though SPF was similar.

Model	1 Cell Type		2 Cell Types	
	**Type I Error Rate**	**Power**	**Type I Error Rate**	**Power**
SPOT	0.060	0.975	0.062	0.969
FunSpace	0.122	0.855	0.153	0.837
SPF	0.062	0.975	0.066	0.959
Choose 'Best' Radius	0.417	0.999	0.478	0.998
Choose 'Worst' Radius	0.000	0.037	0.000	0.007

Table 5: Type I error rates and power for a survival outcome with a single image per person and cell locations simulated using a Strauss process.

2.5 Small Sample Size

Finally, we consider the performance of SPOT on simulated data with a smaller sample size. Here we simulated $M = 30$ samples with a single image per person. Images were simulated using a multivariate Gaussian distribution, as discussed in the main manuscript and illustrated in Figure 2. We simulated a survival outcome as described in the main manuscript. With a reduced sample size, the power across methods dropped and type I error rates increased. Among the methods, SPOT provided among the best type I error control and power, though FunSpace was more powerful with two cell types.

Model	1 Cell Type		2 Cell Types	
	**Type I Error Rate**	**Power**	**Type I Error Rate**	**Power**
SPOT	0.074	0.444	0.068	0.395
FunSpace	0.225	0.442	0.284	0.499
SPF	0.082	0.344	0.068	0.281
Choose 'Best' Radius	0.467	0.848	0.530	0.828
Choose 'Worst' Radius	0.000	0.000	0.000	0.000

Table 6: Type I error rates and power for a survival outcome with a single image per person and a reduced sample size (M=30 samples).

References

[1] Amber M Johnson, Jennifer M Boland, Julia Wrobel, Emily K Klezcko, Mary Weiser-Evans, Katharina Hopp, Lynn Heasley, Eric T Clambey, Kimberly Jordan, Raphael A Nemenoff, et al. Cancer cell-specific major histocompatibility complex ii expression as a determinant of the immune infiltrate organization and function in the nsclc tumor microenvironment. *Journal of Thoracic Oncology*, 16(10):1694–1704, 2021.

[2] Yoav Benjamini and Yosef Hochberg. Controlling the false discovery rate: a practical and powerful approach to multiple testing. *Journal of the Royal statistical society: series B (Methodological)*, 57(1):289–300, 1995.

[3] Adrian Baddeley, Ege Rubak, and Rolf Turner. *Spatial point patterns: methodology and applications with R.* CRC press, 2015.

[4] Souvik Seal, Brian Neelon, Peggi Angel, Elizabeth C O'Quinn, Elizabeth Hill, Thao Vu, Debashis Ghosh, Anand Mehta, Kristin Wallace, and Alexander V Alekseyenko. Spaceanova: Spatial co-occurrence analysis of cell types in multiplex imaging data using point process and functional anova. *bioRxiv*, 2023.

[5] Nicolas P Canete, Sourish S Iyengar, John T Ormerod, Heeva Baharlou, Andrew N Harman, and Ellis Patrick. spicyr: spatial analysis of in situ cytometry data in r. *Bioinformatics*, 38(11):3099–3105, 2022.
